# Residual Prostatic Tissue Mimics Anterior Wall Carcinoma of the Bladder: A Rare Complication of the Holmium Laser Enucleation of the Prostate (HoLEP)

**DOI:** 10.7759/cureus.72984

**Published:** 2024-11-04

**Authors:** Burtac Talha Akkurt, Umut Arslan, Serkan Akan

**Affiliations:** 1 Department of Urology, University of Health Sciences, Fatih Sultan Mehmet Research and Training Hospital, Istanbul, TUR

**Keywords:** benign prostate enlargement, bladder cancer, holmium laser enucleation of the prostate (holep), radiological misdiagnosis, transurethral resection of prostate

## Abstract

The holmium laser enucleation of the prostate (HoLEP) is a widely accepted and reliable treatment for benign prostatic hyperplasias. In developing countries where HoLEP surgery has only recently been introduced, its popularity is steadily increasing. In our case, the patient's history of HoLEP and the image of the irregular mass extending from the anterior bladder wall into the lumen were initially misdiagnosed as bladder cancer. In our review of the English literature, we were not able to find any cases similar to ours, in which the residual prostatic tissue following a HoLEP mimicked a bladder cancer.

## Introduction

The holmium laser enucleation of the prostate (HoLEP) is a reliable treatment option for benign prostate hyperplasias (BPHs), regardless of the prostate size, and is becoming increasingly popular worldwide [1]. Recent advances in enucleation techniques offer improved outcomes and reduce complication rates compared with traditional resection methods like the transurethral resection of the prostate (TURP) [2]. Postoperatively, some patients may experience complications such as bladder neck contracture, urethral stricture, or other complex issues [3]. Additionally, current surgical procedures may not achieve satisfactory symptom relief or may fail to prevent the recurrence of bladder outlet obstruction over time, potentially necessitating a surgical retreatment. However, HoLEP is one of the procedures with a low reoperation rate [3]. 

The prostatic tissue, as indicated in some studies, may mimic bladder cancer due to some factors such as prostate cancer intruding into the bladder, prostate cancer metastasis, prostate cancer recurrence, or an ectopic prostatic tissue [4-7].

In our case, the atypical anterior indentation of a large-sized residual prostatic tissue following HoLEP surgery was misleading on imaging studies, as it resembled primary bladder cancer.

## Case presentation

A 59-year-old male smoker with a history of a HoLEP procedure due to benign prostatic obstruction and urinary retention, which was performed at a different regional facility two years ago, presented with complaints of intermittent macroscopic hematuria over the past month and persistent lower urinary tract symptoms (LUTS). After the HoLEP operation, there was a partial regression in LUTS complaints, but the symptoms recurred one year ago. The prostate-specific antigen (PSA) level was 3.6 ng/mL. The clinical examination and additional laboratory tests did not reveal any significant findings (Table 1).

**Table 1:** Laboratory investigations ALT: alanine transaminase, AST: aspartate transaminase, PSA: prostate-specific antigen, KFT: kidney function test, Na: sodium, K: potassium, CBC: complete blood count, Hb: hemoglobin, WBC: white blood cell, RBC: red blood cell, PT: prothrombin time, INR: international normalized ratio, APTT: activated partial thromboplastin time.

Test	Observed value	Reference range
ALT	23U/L	7-56 U/L
AST	17 U/L	5-40 U/L
PSA	3.6 ng/mL	0-4 ng/mL
KFT		
Blood urea nitrogen	11.8 mg/dL	7-20 mg/dL
Serum creatinine	0.8 mg/dL	0.6-1.3 mg/dL
Na	142 mmol/L	135-145 mmol/L
K	4.2 mmol/L	3.5-5.0 mmol/L
CBC		
Hemoglobin	12.6 g/dL	12.1-15.1 g/dL
WBC	4.2 x 10^3^/µL	4.0-11.0 x 10^3^/µL
Platelets	293 x 10^3^/µL	150-450 x 10^3^/µL
RBC	4.1 x 10^3^/µL	4.0-5.0 x 10^6^/µL
Coagulation profile		
PT	11 seconds	10.0-14.0 seconds
INR	1.1	0.8-1.2
APTT	27 seconds	25-35 seconds

**Figure 1:**
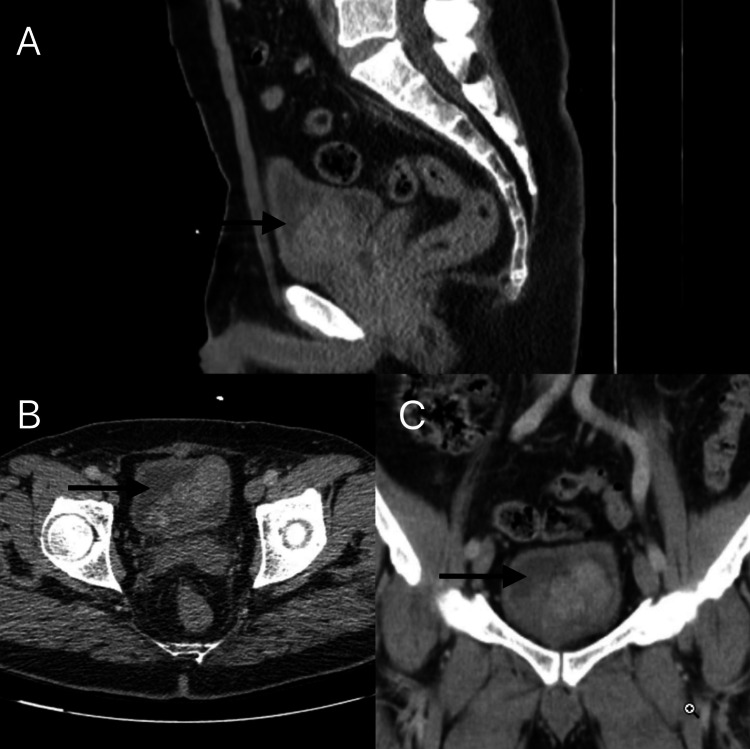
Contrast-enhanced CT of the mass Sagittal (A), axial (B), and coronal (C) slices showing a mass in the bladder lumen (black arrows).

The urinary system ultrasound disclosed an echogenic, hypervascular lesion approximately 70 x 50 mm in size with an irregular shape. This lesion was located on the anterior wall of the bladder. Subsequently, the contrast-enhanced computer tomography (CT) scan demonstrated a lobulated, contrast-enhancing, vascularized, solid, heterogeneous mass approximately 70 x 37 x 53 mm in size, extending from the anterior wall of the bladder into the bladder lumen and the prostatic fossa (Figure 1). 

**Figure 2:**
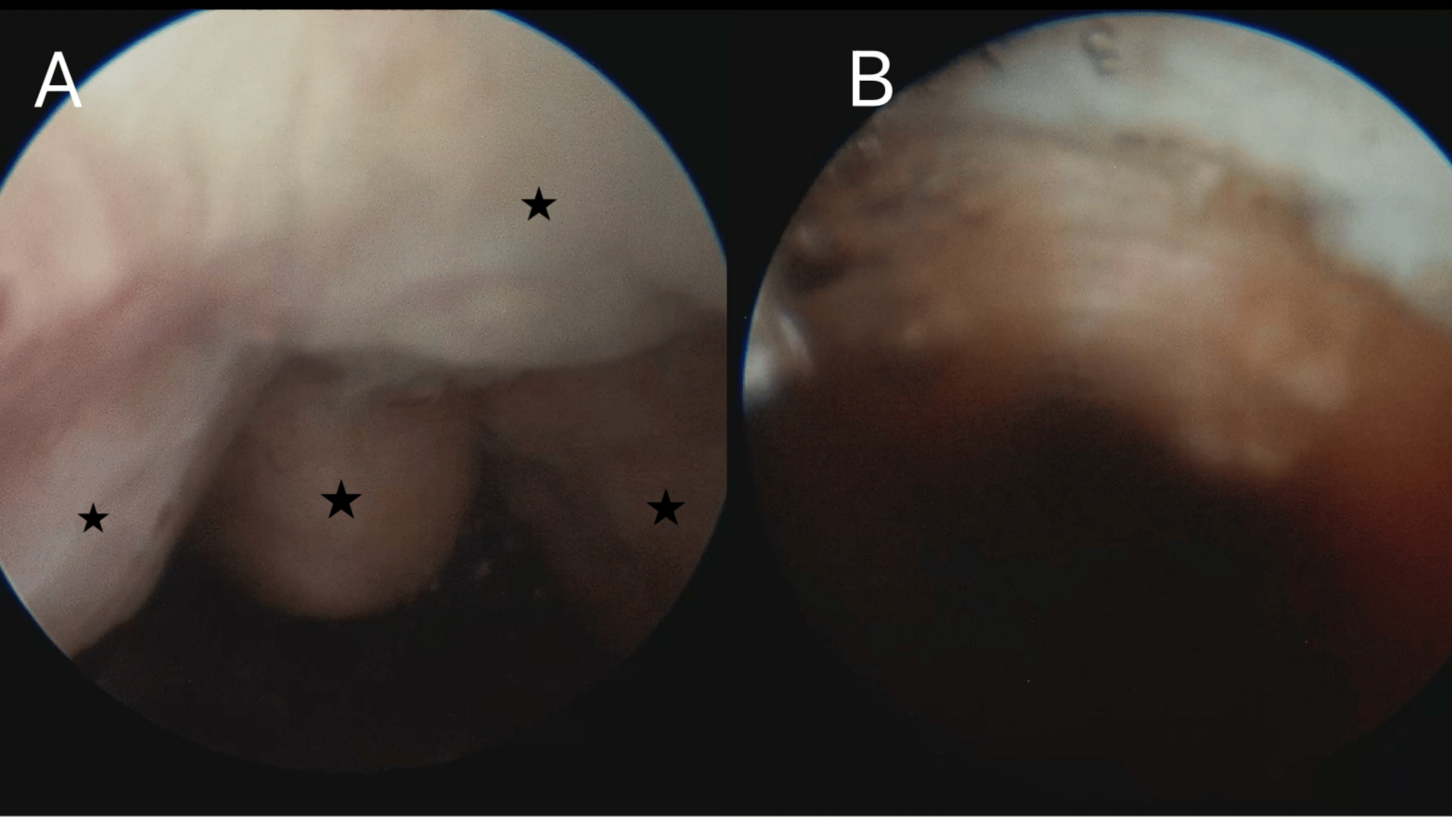
Cystoscopic appearance Irregular prostatic adenomas (black stars) protruding anteriorly into the bladder (A) and following monopolar TURP (B). TURP: transurethral resection of the prostate.

The cystoscopy revealed irregular prostatic adenomas obstructing the prostatic fossa and the protrusion prominently into the bladder from the 11 to 1 o'clock positions, extending along the anterior wall of the bladder (Figure 2A). Apart from the increased trabeculation, no bladder tumors or other pathological findings were observed. The complete resection of the residual prostatic tissue of 80 cc was performed via the monopolar TURP in 90 minutes (Figure 2B).

The histopathological examination revealed adenomatous and myxomatous hyperplasias. Immunohistochemical analysis showed a negative AMACR, positive HMWCK, and positive P63 staining.

## Discussion

Since its development in the 1990s, the HoLEP has benefited from advancements in laser technology, morcellation devices, and surgical techniques, providing a safe, effective, lower-morbidity endoscopic BPH treatment [8]. In their review, Shvero et al. suggest that the HoLEP represents the new gold standard for BPH treatment due to its lower complication rates, shorter catheterization and hospitalization times, comparable safety and efficacy to other procedures, and applicability in patients on anticoagulants and with varying prostate sizes [1].

However, the disadvantage of a HoLEP is that it has a steep learning curve. In the study conducted by Bae et al. on the learning curve of the HoLEP (a single-center experience), a significant reduction in the TURP conversion and mucosal injury rates was observed after the first 30 cases, along with a notable increase in enucleation and morcellation efficiencies [9]. Even when performed by experienced surgeons who have completed the learning curve, it is not devoid of complications and sequelae [10]. Therefore, modified HoLEP techniques have been developed in the last 20 years to facilitate the application technique and reduce complication rates [11]. 

One of the controversial aspects of our report is that the initial HoLEP procedure was performed by a former physician, and the exact reason for the presence of the residual prostatic tissue remains unclear. It is possible that the procedure was conducted by an inexperienced physician who had not yet completed the learning curve, potentially leading to complications. Alternatively, the procedure might have been interrupted by some factors such as a serious adverse effect related to the anesthesia, a technical difficulty, a malfunctioning equipment, or a significant change in the vital signs associated with comorbidities, which can be encountered in any surgical intervention. The analysis of the patient's previous operation report does not specify which HoLEP technique was used or whether any of the abovementioned difficulties were encountered. However, the enucleation of a giant prostatic tissue invading the anterior bladder wall may be challenging even for an experienced surgeon.

It is not uncommon for the prostatic tissue that grows into the bladder to be misdiagnosed as bladder cancer. One of the causes, an ectopic prostatic tissue, is a rare condition with fewer than 50 cases reported in the literature, and its most common location is the bladder trigone [12]. A retrospective study conducted in China demonstrated that up to 3% of the patients diagnosed with prostate cancer were initially misdiagnosed with bladder cancer due to their tumors originating from the prostate base and intruding into the bladder. However, none of these cases involved intrusion into the anterior wall of the bladder [7]. Although these prostatic tissues are typically observed in the posterior wall of the bladder or the trigonal region, prostate cancer may rarely metastasize to the anterior wall of the bladder [13].

In our case, the patient's previous history of undergoing a HoLEP procedure and the imaging findings of an irregularly shaped, hypervascular, contrast-enhancing solid mass extending from the anterior bladder wall into the bladder lumen raised the initial suspicion of bladder urothelial carcinoma or adenocarcinoma. However, the cystoscopy and resection followed by the immunohistochemical analysis confirmed that this mass was a residual prostatic tissue from the previous HoLEP procedure.

## Conclusions

In patients with a history of a HoLEP surgery, residual prostatic tissues may persist and exhibit atypical anterior protrusion into the bladder, which could be misdiagnosed as bladder cancer on the imaging. Surgeons should be cognizant of the potential for such complications, particularly in regions where the HoLEP is newly introduced and the learning curve for the procedure is still being navigated. Moreover, this case underscores the importance of experienced surgeons in minimizing postoperative complications, as incomplete enucleation or technical challenges can result in complex sequelae, as observed in this instance. Greater awareness of this rare but significant issue may contribute to improved patient outcomes and help prevent misdiagnoses in both established and emerging healthcare settings.
